# The American cranberry: first insights into the whole genome of a species adapted to bog habitat

**DOI:** 10.1186/1471-2229-14-165

**Published:** 2014-06-13

**Authors:** James Polashock, Ehud Zelzion, Diego Fajardo, Juan Zalapa, Laura Georgi, Debashish Bhattacharya, Nicholi Vorsa

**Affiliations:** 1 USDA, Agricultural Research Service, Genetic Improvement of Fruits and Vegetables Lab, 125A Lake Oswego Rd., Chatsworth, New Jersey 08019, USA; 2 Department of Ecology, Evolution and Natural Resources, Rutgers University, 59 Dudley Rd., New Brunswick, New Jersey 08901, USA; 3 Department of Horticulture, University of Wisconsin, 1575 Linden Drive, Madison, Wisconsin 53706, USA; 4 USDA, Agricultural Research Service, Vegetable Crops Research Unit, 1575 Linden Drive, Madison, Wisconsin 53706, USA; 5 P.E. Marucci Center for Blueberry and Cranberry Research, 125A Lake Oswego Rd., Chatsworth, New Jersey 08019, USA; 6 Current address: American Chestnut Foundation, Meadowview Research Farms, 29010, Hawthorne, Dr., Meadowview, Virginia 24361, USA; 7 Department of Plant Biology and Pathology, Rutgers University, 59 Dudley Rd., New Brunswick, NJ 08901, USA

**Keywords:** *Vaccinium macrocarpon*, Ericaceae, Transcriptome, COSII, Polyphenolics, Resistance genes, SSRs, SNPs, Inbred

## Abstract

**Background:**

The American cranberry (*Vaccinium macrocarpon* Ait.) is one of only three widely-cultivated fruit crops native to North America- the other two are blueberry (*Vaccinium* spp.) and native grape (*Vitis* spp.). In terms of taxonomy, cranberries are in the core Ericales, an order for which genome sequence data are currently lacking. In addition, cranberries produce a host of important polyphenolic secondary compounds, some of which are beneficial to human health. Whereas next-generation sequencing technology is allowing the advancement of whole-genome sequencing, one major obstacle to the successful assembly from short-read sequence data of complex diploid (and higher ploidy) organisms is heterozygosity. Cranberry has the advantage of being diploid (2*n* = 2*x* = 24) and self-fertile. To minimize the issue of heterozygosity, we sequenced the genome of a fifth-generation inbred genotype (F ≥ 0.97) derived from five generations of selfing originating from the cultivar Ben Lear.

**Results:**

The genome size of *V. macrocarpon* has been estimated to be about 470 Mb. Genomic sequences were assembled into 229,745 scaffolds representing 420 Mbp (N50 = 4,237 bp) with 20X average coverage. The number of predicted genes was 36,364 and represents 17.7% of the assembled genome. Of the predicted genes, 30,090 were assigned to candidate genes based on homology. Genes supported by transcriptome data totaled 13,170 (36%).

**Conclusions:**

Shotgun sequencing of the cranberry genome, with an average sequencing coverage of 20X, allowed efficient assembly and gene calling. The candidate genes identified represent a useful collection to further study important biochemical pathways and cellular processes and to use for marker development for breeding and the study of horticultural characteristics, such as disease resistance.

## Background

The American Cranberry (*Vaccinium macrocarpon* Ait.) is native to North America and is a member of the Ericaceae (the heath family). Cranberry fruit was collected from the wild by American Indians and used for a variety of purposes including as a preservative of fish and meat and medicinally as a poultice for dressing wounds [1]. The crop was first domesticated in the early 1800s on Cape Cod, Massachusetts. Today, cranberries are grown primarily in Wisconsin, Massachusetts, New Jersey, in the Pacific Northwest in Oregon and Washington, and in five provinces in Canada. Outside of North America, *V. macrocarpon* is cultivated in parts of Europe and Chile. In 2012, US cranberry production was 804 million pounds, valued at over $385 million (USDA-NASS, 2012). The American cranberry and other species of *Vaccinium*, such as highbush blueberry (*V. corymbosum* L.) are known for their phytochemicals that can benefit human health [2,3]. Cranberry, in particular, is high in polyphenolic antioxidants [4,5], helps prevent urinary tract infections [6-8], has some anticancer properties [9,10], and may help prevent oral caries [11,12] among other health benefits.

*V. macrocarpon* is taxonomically placed in the core eudicots, a diverse group of angiosperms. It is a member of the asterid clade, which contains the orders Cornales and Ericales [13]. The order Ericales is quite diverse and includes 22 families. Key economically important plants in the Ericales include tea (*Camellia sinensis*) and edible fruits such as kiwi (*Actinidia deliciosa*) and persimmon. The Ericaceae is an important family in the Ericales. Members of this family tend to thrive in nutrient-poor acid soils. The Ericaceae includes 126 genera and about 4000 species [14]. Despite the importance of Ericaceous plants such as blueberry, huckleberry, bilberry, cranberry and rhododendron, whole-genome sequence data for this group are currently lacking. Cranberry is diploid (2*n* = 2*x* = 24) and self-fertile, allowing generation of inbred lines. Cranberry can hybridize with, and give rise to fertile offspring, when crossed to related species such as *V. oxycoccus* L. [15], producing unique populations segregating for a host of horticultural and biochemical characteristics. A major characteristic important for sustainability is disease resistance. Cultivated cranberry is susceptible to a variety of fungal pathogens [16-18]. In fact, fruit rot is the most significant problem in cranberry production in the Northeastern U.S. where, in the absence of an appropriate fungicide regime, the entire crop can be lost [16]. Disease resistance in plants is complex, involving many different pathways and mechanisms. Whereas some of the resistance genes identified in various plant species impart resistance to specific pathogens, others have been implicated in broad-spectrum resistance [19-22]. Identification of putative resistance genes and their mapping for marker-assisted breeding (MAB) would be facilitated by whole-genome sequence analysis. Whole-genome sequence data can be used for many other purposes such as identification of key biosynthetic pathway genes, genotyping, structure-function studies, and evolutionary studies.

The American cranberry has several characteristics including compact size, a relatively short generation interval (for a woody perennial), ease of asexual propagation (via stolons), diploidy, self-fertility, and moderate genome size, that make it suitable for use as a model system, specifically for temperate woody perennial plants species. We present here the results of gene discovery utilizing whole-genome and transcriptome sequence data from an inbred line of American cranberry. The data were analyzed to validate the taxonomic position of cranberry, to identify synteny with other representative sequenced plant species, to predict the majority of the encoded genes, and to identify genes potentially associated with disease resistance.

## Results and discussion

### Genome and transcriptome sequencing and assembly

We sequenced the genome of *V. macrocarpon* using the Illumina GAIIx sequencer. A total of 60 million paired-end reads (2×150 bp) were generated, corresponding to 8.8 Gbp of genomic data. The assembly resulted in 231,033 contigs (N50 = 4,214 bp). Scaffolding slightly reduced the number of contigs to 229,745 and raised the N50 to 4,237 bp. The assembled size was 420 Mbp, with an average sequencing coverage of 20X, comparable to that reported for *Vitis vinifera* (487 Mbp, http://plantgdb.org). The cranberry genome size was estimated to be about 470 Mbp [23]. We believe that this genome size is reasonably accurate (even at the relatively low average coverage) because 93% of the individual sequence reads mapped to the assembly (at 90% similarity over 85% of the read length). This suggests that the assembly includes the vast majority of the sequenced data. Furthermore, repeated regions of the assembly (see below) did not show extreme coverage values (i.e., they varied between 20-35×, similar to overall average genome coverage) suggesting that we are not underestimating genome size due to the co-assembly of distinct repeats into single contigs. Evaluation of the completeness of the *V. macrocarpon* draft genome assembly was done using CEGMA [24,25] and showed that 377 (82%) out of the 458 Core Eukaryotic Genes (CEG) were present in the assembly. Further analysis of the data using the 248 highly conserved CEGs showed that 212 (85.5%) were present in the *V. macrocarpon* assembly with 137 (55.2%) being complete, and the remaining 75 being partial.

A total of 63.6 million reads of mRNA-Seq (2×100 bp) data were generated on the Illumina GAIIx sequencer and assembled into 90,547 cDNAs longer than 300 bp and with average coverage >5X. The cDNA set was aligned to the genome assembly resulting in 48,271 cDNAs (53.3%) mapping with 98% identity. These assemblies and the mRNA-Seq reads were used to guide the gene prediction program.

The genome and transcriptome data have been deposited in NCBI as BioProject (PRJNA245813) and BioProject (PRJNA246586) respectively.

### Transposable elements

Genome-wide sequence analysis has demonstrated that transposable elements (TE) of different types are widespread in eukaryotes. In flowering plants, transposons can account for large percentages of the total genome. Even in the relatively small (~150 Mbp) *Arabidopsi*s genome, transposons comprise about 18.5% percent of the genome. The larger (~2,700 Mbp) maize genome contains greater than 70% of the genome as transposons. It is becoming clear as more plant genomes are analyzed that transposons contribute to the size and diversity of plant genomes. Although we found representatives of most classes of known transposons, the Class 1 retrotransposons of the LTR (long terminal repeat) type are the most common, as has been found in other plants (Table 1). Surprisingly though, despite the fact that the cranberry genome (420-470 Mbp) is about 3 times that of *Arabidopsis*, the percentage of transposons in cranberry was found to be only about 5.6%. It is unclear why the percentage in cranberry might be so low. Of this 5.6%, it would be instructive to determine the percentage that might be active. A lack of active transposition would limit expansion of the elements in the genome. In maize, active transposition sometimes gives rise to obvious phenotypic changes. Some of these, such as color change in the aleurone layer of kernels, led to their discovery [26,27]. These types of changes, such as variegated leaves or color variation in the fruit epidermis, have not been observed in cranberry in cultivars that have been domesticated for over 100 years, e.g., ‘Early Black’ and ‘Ben Lear’, as is common in apple [28]. As such, cranberry lacks the phenotypic evidence of active transposition. However, although the Class II TE represented only about 1% of the genome (Table 1), similar to apple and cucumber, the hobo-Activator represented 0.51% of the cranberry genome with a copy number 13,254. Class II TEs are well recognized as promoting chromosomal rearrangements, including reciprocal translocations [29]. Reciprocal translocation heterozygotes have been identified in cranberry [30,31] suggesting Class II TEs may be or have been active in cranberry.

**Table 1:** Transposable elements in the cranberry genome

	**Cranberry**	**Cucumber**	**Apple**	**Grape**	***Arabidopsis***	**Rice**	**Melon**	**Maize**
	**% of genome**	**% of genome**	**% of genome**	**% of genome**	**% of genome**	**% of genome**	**% of genome**	**% of genome**
**Transposable Elements**	39.53	14.80	42.40	21.50	18.50	39.60	24.10	84.20
**Class I: retrotransposons**	10.14	6.9	37.6	19.4	7.5	25.9	12.16	75.60
LTR/Copia	3.50	-	5.5	4.8	1.4	2.5	10.43	23.70
LTR/Gypsy	3.72	-	25.2	14.0	5.2	12.0		46.40
SINE	0.45	-	-	-	-	0.5	0.01	-
LINE	2.20	-	6.5	0.6	0.9	0.8	1.74	1.00
Others	0.27	-	0.4	-	-	-	-	-
Unclassified	-	-	-	-	-	10.1	11.64	4.50
**Class II: DNA transposons**	5.34	0.8	0.9	1.4	11.0	13.7	1.24	8.60
Hobo-Activator	2.51	-	0.3	0.8	0.3	0.5	-	1.1
Tc1-IS630-Pogo	0.10	-	-	-	0.1	-	-	-
Tourist/Harbinger	0.25	-	-	-	0.2	1.5	-	1.0
Others	2.48	-	0.6	0.6	9.9	7.4	-	6.5
Unclassified		-	-	-	0.5	4.3	-	-
**Unknown**	24.04	7.1	3.9	0.7	-	-	-	-

The percent of the genome for each type are shown relative to other selected plant species.

Cranberry is reported to have gone through a severe genetic bottleneck, possibly during the Pleistocene [32-34]. As a result, wild populations were found to lack phenotypic variability and have relatively low genetic heterogeneity [32,34]. Cranberry is self-fertile and the proposed genetic bottleneck would result in at least some level of inbreeding. In our studies, a 5^th^ generation inbred line of cranberry was selected for sequencing and it is not known how this level of inbreeding might affect the transposon complement in the cranberry genome. In maize, where inbred lines are used routinely for breeding, high levels of transposons are maintained.

### Gene prediction

Annotation of the *V. macrocarpon* genome assembly was done using AUGUSTUS [35] which was trained to be *V. macrocarpon* specific (see Methods). A set of 43,502 genes was predicted, out of which 1,880 genes had alignments larger than 30% to a TE protein database [36]; these genes were filtered out. The remaining 41,622 genes were used as query for a BLASTP (*e*-value <1E-6) search against the NCBI NR database. Combining the genes that had a BLAST hit (28,226; 68%) with those that had not and were larger than 100 amino acids in length (8,138; 19.5%) resulted in a set of 36,364 genes that was used for further downstream analysis. The average coding sequence size was calculated to be 837 bp and the mean number of exons per gene was 3.1; the gene density was calculated to be 8.3 genes per 100 Kbp. Comparison of *V. macrocarpon* mRNA-Seq data to the set of 36,364 gene models showed that 36% (13,170) of the genes had transcriptome evidence supporting the exons.

To determine whether some of the gene models in *V. macrocarpon* may be artifacts associated with predicted regions in repeats, we re-ran the gene prediction using an assembly that had been masked for de novo repeats (see Methods). This procedure resulted in the prediction of 31,867 gene models. To test whether the prediction made without masking repeats was significantly different, we compared the two sets of gene predictions (i.e., 36,364 vs. 31,867) to each other. Using BLASTP with a cut-off < 1E-10 for this comparison, 97% of the new models were found to be present in the more extensive set of predictions. Given this evidence for the accuracy of the gene prediction with respect to potential biases introduced by repeats, we used the larger set of gene models for downstream analyses.

### Taxonomic assessment

**Figure 1:**
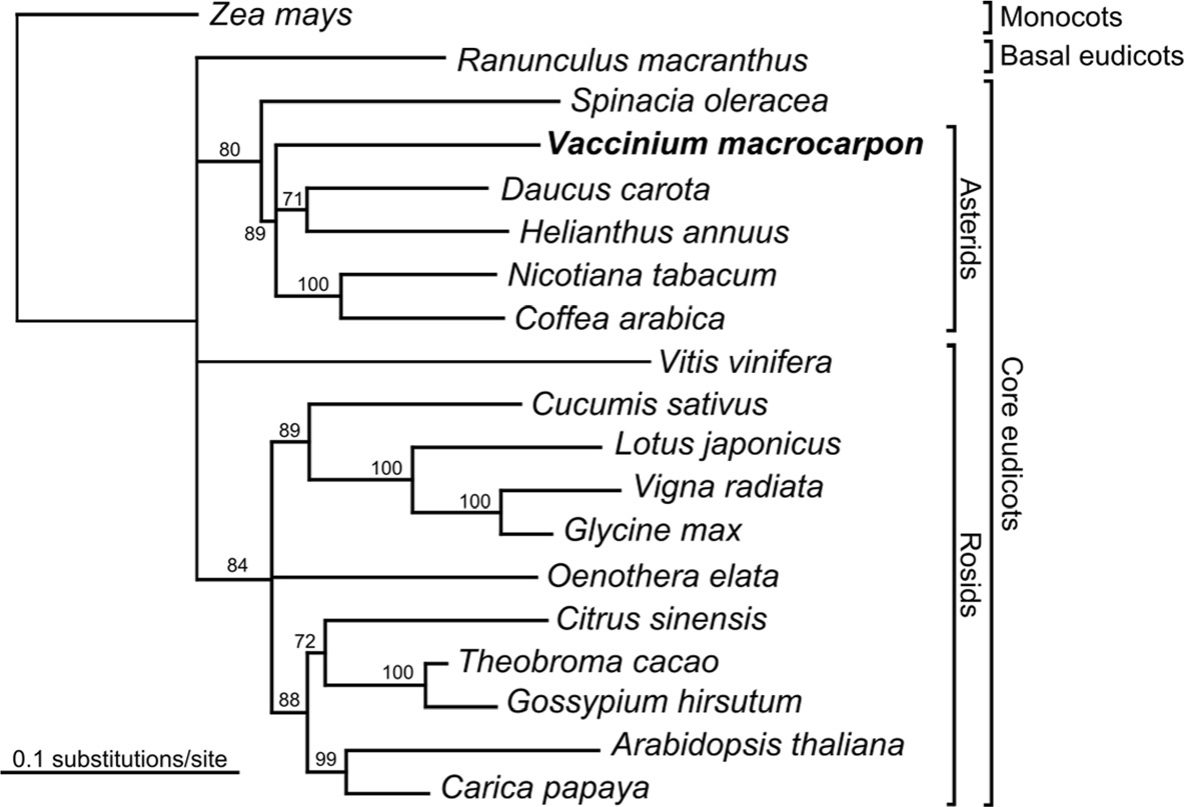
**Multi-gene phylogenetic tree of cranberry and 18 other plant species.** This maximum likelihood (RAxML) tree was built using two plastid (atpB, rbcL) and one mitochondrial (matR) gene sequences which were concatenated and aligned, using MUSCLE (EMBL-EBI), with those of 18 other plant species including Rosids, Asterids, monocots and a basal eudicot. The results of 100 bootstrap replicates are shown at the nodes of this unrooted phylogeny. Branch lengths are proportional to divergence (see scale).

To verify the taxonomic placement of cranberry, two chloroplast- and one mitochondrial-encoded protein(s) were concatenated and aligned with those of 18 other plant species including four asterids (*Coffea arabica, Daucus carota, Helianthus annuus* and *Nicotiana tabacum*), 11 rosids (*Arabidopsis thaliana*, *Carica papaya*, *Citrus sinensis*, *Cucumis sativus*, *Gossypium hirsutum*, *Glycine max*, *Lotus japonicus*, *Oenothera elata*, *Theobroma cacao*, *Vigna radiata* and *Vitis vinifera*), one basal eudicot (*Ranunculus macranthus*), and one monocot (*Zea mays*). The complete chloroplast genome of cranberry has been published [37]. A maximum likelihood (RAxML) tree was built and the results of 100 bootstrap replicates were used to determine branch support in an unrooted phylogeny (Figure 1). Most branches in the tree were well supported and of the species compared, cranberry was clearly located in the asterid group.

More specifically, cranberry is in the Ericaceae (heath family) in the section *Oxycoccus*. Within this section are at least two species, *Vaccinium macrocarpon* Ait. (large-fruited American cranberry) and *V. oxycoccus* L. (European cranberry) [38]. Although *V. oxycoccus* occurs at the diploid, tetraploid and hexaploid levels, and older literature often splits some of these into different species, the section *Oxycoccus* is fairly well defined. In contrast, section *Cyanococcus* contains many blueberry species, the boundaries of which are less clearly defined [39]. Traditional methods coupled with molecular data such as the chloroplast *rbcL* and *matK* genes and nuclear sequences such as ITS, have been used to study phylogenetic relationships within the blueberry tribe (Vaccinieae) [40] and for wider placement in the Ericaceae [14]. The use of the *matR* mitochondrial gene has been proposed for phylogenetic studies in rosids, but not in the order Ericales [41]. As the taxonomy of higher plants continues to advance, it is likely that more molecular data will be needed and utilized. Thus, the genomic data presented here can be mined for various gene sequences or molecular markers that can be used for this purpose.

### Conserved orthologous set (COSII) markers

Single or low copy nuclear DNA markers are useful for phylogenetic studies and comparative genomic analyses. A COS is defined as a gene conserved in sequence and copy number that shares a common ancestor by descent, while paralogs are duplicated sequences resulting in gene duplications or polyploidization events [42-45]. COS markers have been successfully developed and used for phylogenetic inference among species in the Solanaceae and Poaceae families [44,46,47].

The determination of COS markers in *Vaccinium macrocarpon* is of importance to resolve the phylogenetic relationship of the cranberry and wild relative species in the *Ericaceae* family.

The comparison between *Arabidopsis* and sunflower transcriptomes yielded 110 single copy orthologous genes, while the comparison against *Arabidopsis* and lettuce detected 171 genes. There were 41 putative COSII markers in common between the two datasets, of which 35 had known function (Additional file 1: Table S1).

### Microsatellite detection

**Figure 2:**
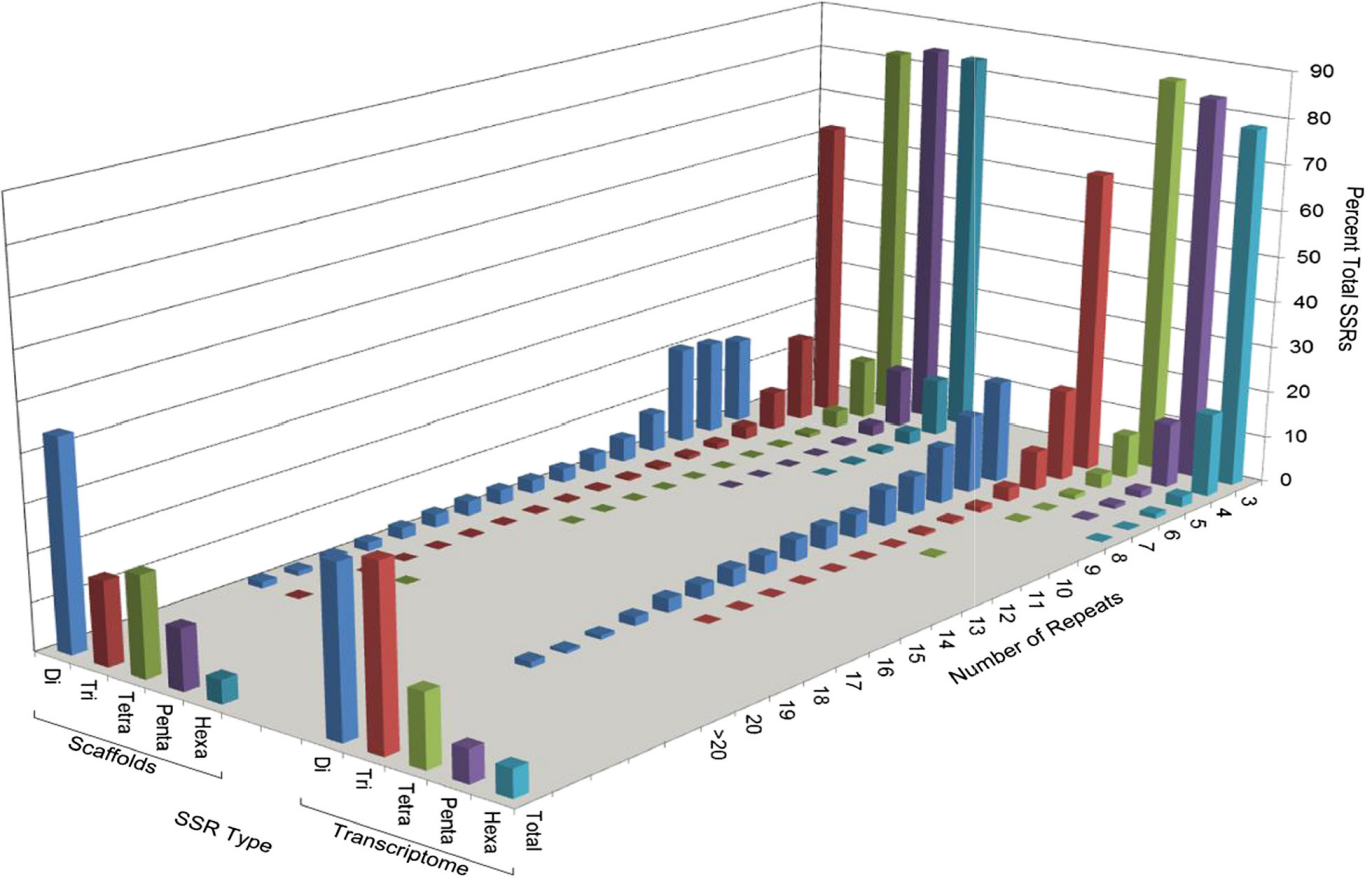
**Microsatellite (Simple Sequence Repeat; SSR) markers.** Occurrence and distribution in the cranberry genome and unigene (transcriptome) sequences.

A total of 159,394 perfect SSRs were detected in the sequenced and assembled genome. A set of 150,628 and 8,766 SSRs corresponded to 86,884 assembled genome scaffolds and 7,772 unigene sequences, respectively. The most frequent motif length repeated in the genome scaffolds corresponded to di-nucleotides (44%) (Figure 2). The maximum repeat length was 87 and the average length of the SSRs was 16 nucleotides. The nuclear scaffolds showed di- and tetra-nucleotides to be the most abundant motifs representing 44 and 21% of the SSRs, respectively. The GA and AAAT motifs were the most abundant corresponding to 16.5 and 3.6% of the total detected SSRs, respectively. For the microsatellites located in the unigene sequences, tri- and di-nucleotides were the most abundant motifs accounting for 37% and 35%, respectively, and the longest SSR was 66 nucleotides. The most frequent di-, tri-, and tetra-nucleotide motifs were GA, AAG and AAAT accounting for 15, 3.5 and 1.6%, respectively, of the transcriptome SSRs. This is the first whole genome microsatellite search in cranberry and provides the largest data set available until now of easy-to-use markers for molecular-based studies in cranberry and other *Vaccinium* crops.

### SNP distribution

We used a 5^th^ generation inbred accession (CNJ99-125-1) of cranberry to lower heterozygosity and facilitate assembly. Assuming ‘Ben Lear’ has a coefficient of inbreeding (F) = 0, the estimated heterozygosity of the clone sequenced would be 3.1%. However, ‘Ben Lear’ was reported to have 6 of 12 SSR loci being homozygous, the highest among cultivars analyzed [48], suggesting F > 0. The bivariate SNP distribution showed a total of 602,000 SNPs that occur at a minimum frequency of 20% across reads that provide >10X genome coverage. The total number of SNPs detected represents a level of heterozygosity of about 0.14%, in our 420 Mbp assembly. To determine the ‘starting’ level of homozygosity, we compared these results with those from the ‘Ben Lear’ parent. Using the cranberry transcriptome as reference to search for common SNPs, comparisons between ‘Ben Lear’ (parent) and CNJ99-125-1 yielded 25,803 versus 20,180 SNPs, respectively (Table 2). CNJ99-125-1 possessed fewer Multi-Nucleotide Variants (541) and Single-Nucleotide Variants (16,968), than ‘Ben Lear’ (790 and 21,084, respectively). Additionally, CNJ99-125-1 showed a reduction in indels (insertions = 421 and deletions = 2,250) when compared with ‘Ben Lear’ (insertions = 763 and deletions = 3,166) (Table 2). Overall, CNJ99-125-1 showed a general reduction in variability based on all SNP types observed.

**Table 2:** SNP comparison between ‘Ben Lear’ and inbred accession CNJ99-125-1

**SNP type**	**Ben Lear**	**CNJ99-125-1**
Deletion	3166	2250
Insertion	763	421
MNV	790	541
SNV	21084	16968
**Total**	25803	20180

MNV = Multi-Nucleotide Variants, SNV = Single-Nucleotide Variants.

Many different marker systems have been used for cultivar identification in cranberry including RAPDs [49], SCARs [50], and SSRs [48,51]. SSRs were also used for mapping and QTL analysis [52,53]. Breeding and selection in cranberry, as with most woody perennials, is a lengthy process, requiring at least 3-4 years from seed to flowering progeny. The progeny are then typically cloned and planted in small field plots for evaluation and selection, which might take an additional 3-5 years. The cranberry breeding program would benefit tremendously from molecular markers that can be used for marker-assisted selection (MAS). This approach has gained in popularity in many crop systems [54]. Until very recently, SSRs were the marker of choice for higher plant breeding and although these markers will probably be used for some time, the use of SNPs is likely to become more widespread.

The utilization of SNPs for MAS has been propelled by the tremendous capability of next generation genome sequencing [55,56]. Similar to SSRs, SNPs also have the advantage of being abundant in plant genomes. Many detection methods are available and continue to be developed for SNP detection, including high throughput platforms such as SNP microarrays. Association of certain SNPs with common diseases has already been described in humans [57,58] and this technology is now being applied to plant research. For example, SNPs were used for genome-wide association study of 107 phenotypes in *Arabidopsis*[59]. Many common alleles with major effect were detected, offering many candidates for follow up studies. Although generally limited to major crops and model systems, this technology will expand into specialty crops such as cranberry.

### Transporters

**Figure 3:**
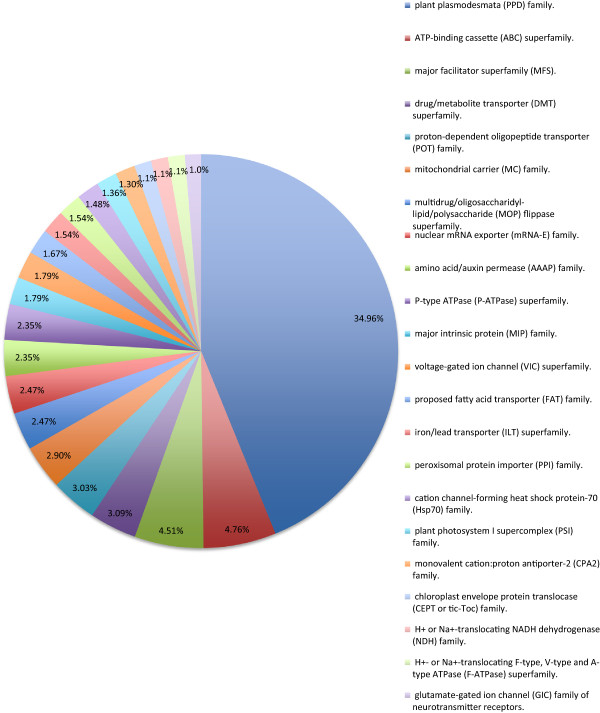
**Transport proteins in the*V. macrocarpon*predicted proteins data set.** Classification was done using 6099 membrane transport protein sequences downloaded from The Transporter Classification Database (TCDB). Shown are the percentages of total for each type (family or superfamily) listed in the legend in order from most to least abundant.

Transport proteins are integral to the movement of materials across membranes - into and out of cells and subcellular compartments, and between cells via plasmodesmata. We identified a total of 1,619 putative transporters in 117 families. Given the fragmented nature of our assembly, we recognize that this number is likely to be an over-estimate because some genes may be fragmented into more than one contig and thus may be mistakenly identified as independent transporter genes. This potential bias is also applicable to all other genes and gene families identified in *V. macrocarpon* (see discussion below). If we consider only those families that are better represented (i.e. that represent more than 1% of the total predicted transport proteins), there are 1,310 in 26 families. By far, the single most abundant group (almost 35% of the total) is the plant plamodesmata family (Figure 3). The relative numbers in each family were similar between grape and cranberry (Table 3). The universality of transporters in eukaryotic systems offers the opportunity for phylogenetic studies and prediction of function [60]. Transporters in plants are also critical to uptake of water and mineral nutrients in the roots. As the global climate changes, plants are increasingly subjected to stresses such as drought, and those grown on more marginal soils may experience salt build up due to irrigation. A better understanding of these proteins will be critical to sustaining agricultural crops.

**Table 3:** The relative numbers of transporters in each family

**Transporter family**	**Cranberry**	**Grape**	**Difference**
Plant plasmodesmata (PPD) family.	34.96%	28.29%	6.67%
Major facilitator superfamily (MFS).	4.51%	2.46%	2.05%
Proton-dependent oligopeptide tranporter (POT) family.	3.03%	1.76%	1.26%
Mitochondrial carrier (MC) family.	2.90%	1.88%	1.02%
Major intrinsic protein (MIP) family.	1.79%	0.98%	0.81%
Nuclear mRNA exporter (mRNA-E) family.	2.47%	1.68%	0.79%
Cation channel-forming heat shock protein-70 (Hsp70) family.	1.48%	0.75%	0.73%
Proposed fatty acid transporter (FAT) family.	1.67%	0.95%	0.71%
Monovalent cation:proton antiporter-2 (CPA2) family.	1.30%	0.61%	0.69%
Amino acid/auxin permease (AAAP) family.	2.35%	1.82%	0.53%
Multidrug/oligosaccharidyl-lipid/polysaccharide (MOP) flippase superfamily.	2.47%	1.99%	0.48%
Plant photosystem I supercomplex (PSI) family.	1.36%	0.95%	0.41%
Voltage-gated ion channel (VIC) superfamily.	1.79%	1.42%	0.38%
Chloroplast envelope protein translocase (CEPT or tic-Toc) family.	1.11%	0.98%	0.13%
P-type ATPase (P-ATPase) superfamily.	2.35%	2.22%	0.12%
Glutamate-gated ion channel (GIC) family of neurotransmitter receptors.	1.05%	1.04%	0.01%
Drug/metabolite transporter (DMT) superfamily.	3.09%	3.18%	âˆ’0.09%
H + -or Na + -translocating F-type, V-type and A-type ATPase (F-ATPase) superfamily.	1.11%	1.36%	âˆ’0.25%
H + -or Na + -translocating NADH dehydrogenase (NDH) family.	1.11%	1.44%	âˆ’0.33%
Iron/lead transporter (ILT) superfamily.	1.54%	2.22%	âˆ’0.68%
ATP-binding cassette (ABC) superfamily.	4.76%	5.46%	âˆ’0.70%
Nuclear pore complex (NPC) family.	0.43%	1.39%	âˆ’0.95%
Nuclear t-RNA exporter (t-Exporter) family.	0.25%	1.42%	âˆ’1.17%
Endoplasmic reticular retrotranslocon (ER-RT) family.	0.49%	1.79%	âˆ’1.30%
Transient receptor potential Ca2+ channel (TRP-CC) family.	0%	1.59%	âˆ’1.59%
Peroxisomal protein importer (PPI) family.	1.54%	6.39%	âˆ’4.84%

Percent shown is based on the total number found in each species.

The difference is between Cranberry vs. Grape.

### Transcription factors

We identified 1,295 transcription factors (TFs), grouped into 80 families, within the *V. macrocarpon* genome (Table 4), compared to 3,667 in apple, 2,705 in *Arabidopsis,* 2,219 in grape, and 3,148 in rice. Thus, the level (1% of the genome) found in cranberry is much lower than in these other plants. The most common TFs in cranberry, as in most plants, are in the MYB superfamily (119), the bHLH (basic helix-loop-helix) family (79) and the AP2/EREBP (APETALA2/ethylene responsive element binding protein) family (74). Together, these accounted for about 21% of the total TFs found. The ZIM family, which is a unique TF containing a GATA-type zinc-finger domain [61], appears to be absent from cranberry, apple, and grape.

**Table 4:** Transcription factors

**TF_family**	**Cranberry**	**Apple**	**Arabidopsis**	**Grape**	**Rice**
MYB	119	428	263	275	252
bHLH	79	244	172	116	196
AP2-EREBP	74	274	166	145	188
C3H	70	148	83	54	109
C2H2	68	313	107	129	124
Orphans	53	136	86	140	216
PHD	52	127	55	70	61
HB	51	169	105	75	137
NAC	41	261	120	81	146
bZIP	37	107	110	50	127
SNF2	33	64	43	35	48
WRKY	32	145	85	64	119
GNAT	29	n.d.	41	32	45
SET	29	68	46	45	34
mTERF	26	n.d.	36	21	29
CCAAT	25	193	72	87	69
GRAS	25	121	36	51	63
Trihelix	23	27	27	62	26
TRAF	22	n.d.	29	19	90
ABI3VP1	21	80	59	36	60
G2-like	20	19	53	35	57
C2C2-Dof	19	58	44	24	36
FAR1	19	n.d.	24	42	129
LOB	19	n.d.	49	45	43
FHA	17	34	19	13	22
MADS	17	152	124	65	87
C2C2-GATA	16	39	37	24	37
AUX/IAA	15	52	35	24	52
TCP	15	58	30	18	25
SBP	14	42	28	19	29
ARF	12	37	35	19	49
HSF	12	56	24	20	40
OFP	12	n.d.	18	11	30
BSD	10	n.d.	12	7	8
HMG	9	23	21	12	20
RWP-RK	9	14	16	7	14
Jumonji	8	29	20	19	17
SWI/SNF-BAF60b	8	n.d.	17	14	10
Transcription Factors					
ARID	7	n.d.	11	10	6
ARR-B	7	16	17	29	12
C2C2-CO-like	7	17	21	6	24
Sigma70-like	7	n.d.	6	6	7
Tify	7	n.d.	26	11	16
BES1	6	24	14	9	6
E2F-DP	6	15	14	7	12
TAZ	6	n.d.	8	3	2
TUB	6	21	15	11	26
CAMTA	5	n.d.	9	4	7
GeBP	5	13	20	3	6
SWI/SNF-SWI3	5	n.d.	5	5	4
zf-HD	5	29	18	17	15
BBR/BPC	4	6	16	3	9
DDT	4	n.d.	5	6	6
GRF	4	n.d.	9	7	13
HRT	4	n.d.	2	1	1
LUG	4	n.d.	7	3	7
Pseudo	4	n.d.	5	5	4
CSD	3	n.d.	4	3	4
Alfin-like	2	n.d.	9	6	9
Coactivator	2	n.d.	7	2	2
CPP	2	n.d.	9	6	11
DBP	2	n.d.	5	4	6
LIM	2	n.d.	10	5	6
PBF-2-like	2	n.d.	4	2	2
PLATZ	2	n.d.	14	9	15
SRS	2	8	13	6	6
C2C2-YABBY	1	n.d.	8	7	7
EIL	1	n.d.	6	4	8
IWS1	1	n.d.	2	1	1
LFY	1	n.d.	1	1	2
MBF1	1	n.d.	3	3	4
MED6	1	n.d.	1	1	1
RB	1	n.d.	1	2	2
Rcd1-like	1	n.d.	3	2	6
SAP	1	n.d.	1	1	0
SOH1	1	n.d.	1	0	2
TIG	1	n.d.	0	0	0
ULT	1	n.d.	2	1	2
VOZ	1	n.d.	3	2	2
ZIM	0	0	23	0	23

The numbers found in cranberry for each family as compared to other selected species.

Transcription factors are important regulators of gene expression. Most transcription factors reported are predicted based on their DNA-binding domain and can be subdivided based on a variety of characteristics such as the number of repeats [62]. Although many have been predicted in plants, experimental characterization to document function is lacking for most. Prediction of biological function is further complicated by the fact that within a family, the origin is probably from gene duplication but divergence has resulted in potentially very different functions [63-65].

### Disease resistance genes

A total of 555 putative R-genes were identified in the *V. macrocarpon* genome and classified using the PRGdb [66] (Table 5). The total number was similar to that found in grape and *Arabidopsis*. Of these, 63 were classified as cytoplasmic proteins that function using the canonical resistance domains like the nucleotide-binding site (NBS), toll/interleukin-1 receptor (TIR) and the leucine-rich repeat (LRR) domains. Moreover, 333 putative *V. macrocarpon* R-genes were found to be transmembrane receptors, of which 289 were classified as receptor-like kinases (RLK) and 44 as receptor-like proteins (RLP). Out of the 555 putative R-genes, 159 were classified as ‘others’, including genes which have been described as conferring resistance through different molecular mechanisms. Within this class 67, 47 and 11 genes showed homology to the rice *Pid2*[67], the tomato *Pto*[68] and the wheat *Lr34* genes, respectively. The best characterized R-genes impart some resistance to biotrophic pathogens. For many small fruits, including cranberry, the majority of the fruit rot pathogens are necrotrophs [16,69]. Using 85 genes implicated in *Arabidopsis* immune responses to necrotrophic pathogens [70] for reference, cranberry was found to have about half as many (42), while grape (60), rice (52), apple (58), and melon (58) were found to have more, but still fewer than *Arabidopsis*. Because the actions of these genes impact susceptibility to pathogens, they may be under positive selection, resulting in sequence divergence that complicates the identification of homologues.

**Table 5:** Disease resistance (R-genes) found in cranberry

**Class**	**Cranberry**	**Grape**	***Arabidopsis***	**Rice**	**Apple**	**Melon**
**CNL** (CC-NB-LRR)	55	60	40	402	181	21
**TNL** (TIR-NB-LRR)	2	19	97	0	224	21
**NL** (NB-LRR)	6	111	11	74	394	10
**RLK** (Ser/Thr-LRR)	289	219	222	394	1265	161
**RLP** (Kinas-LRR)	44	150	91	216	320	110
**Other** (159)						
Pid2	67					
Pto	47	0	1	7		25
Lr34	11					
Bs3	9					
Hm1	8					
MLO	3	17	19	17		15
RTM1	3					
Xa13	3					
Bs3-E	2					
ASC1	1					
At1	1					
Hs1pro-1	1					
Hm2	1					
Hm3	1					
IVR	1					
**Total**	555	576	481	1100	2384	363

The number found in each class as compared to other selected species.

### Mitochondrial genome comparison

**Figure 4:**
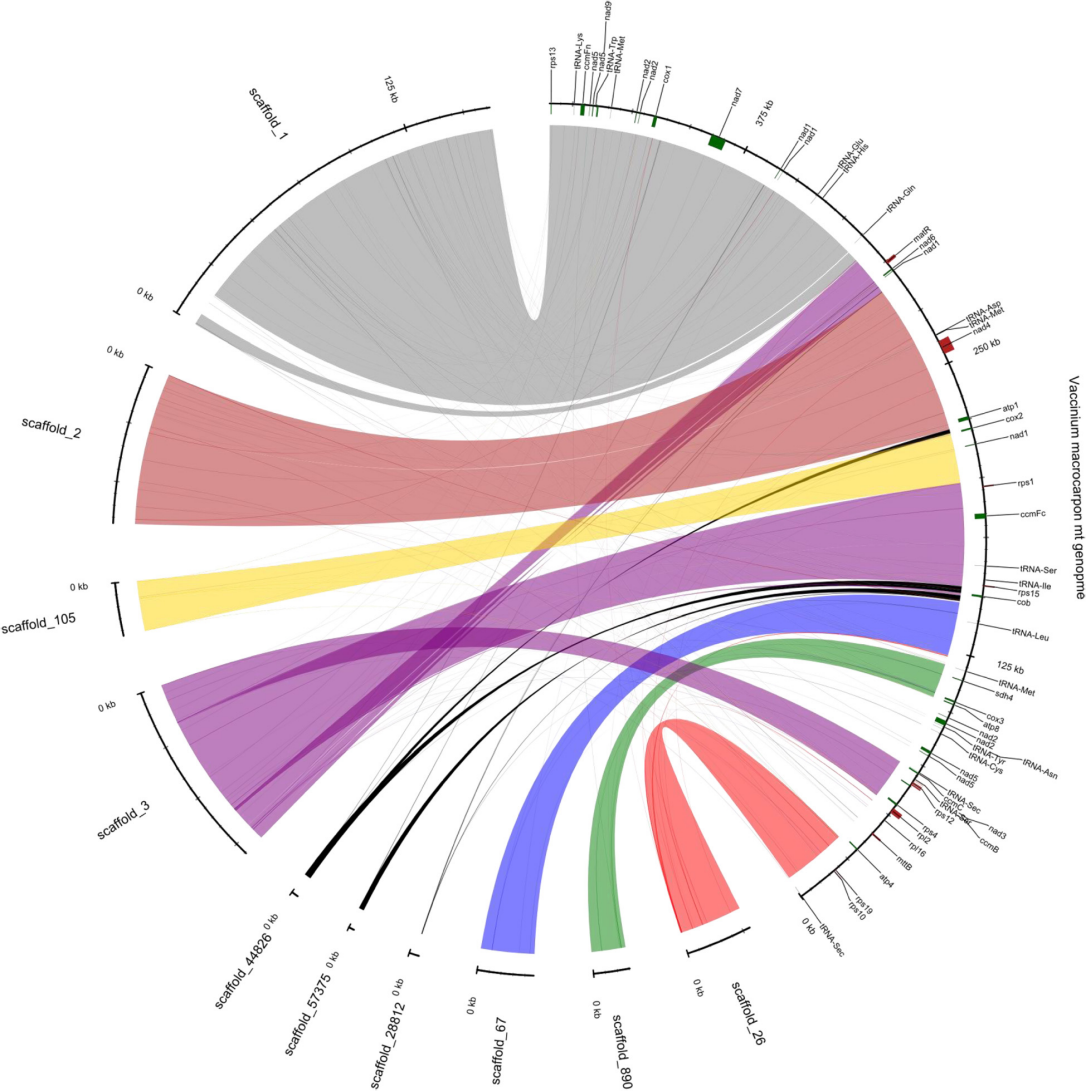
**Mitochondrial scaffolds recovered from the cranberry whole-genome assembly.** Mapped mitochondrial scaffolds (left) against the previously published mitochondrial genome (right). Different colors represent different scaffolds. Scaffolds colored in black mapped in multiple locations.

Intact nuclei are typically prepared for genomic DNA isolation and subsequent high throughput sequencing to reduce organellar contamination. The number of extranuclear genomes per cell varies depending on the species, cell type and age of the tissue. For example, land plants can have high numbers of chloroplasts (and thus many chloroplast genomes) in the leaf cells. Even though precautions were taken to prevent such small genome contamination, some assembled scaffolds showed high similarity to the sequenced mitochondrial genome after mapping using Mummer3. It was not possible to reconstruct these sequences into a single scaffold representing the whole mitochondrial genome (Figure 4). A set of 10 scaffolds showed high similarity to the cranberry mitochondrial genome [71], with one scaffold suggesting a rearrangement or misassembly. Paired end reads that mapped against the mt genome from CNJ99-125-1 were selected for reassembly by using Abyss, obtaining a set of 16 mitochondrial contigs, showing coverage of the complete mitochondrial genome (Additional file 2: Figure S1). The scaffold that showed the rearrangement appeared as three separate contigs, rejecting the possibility of misassembly. Even though the mitochondrial genome was not recovered as a single scaffold, after the comparison against that of ‘HyRed’, we annotated the mitochondrial genes in the assembled scaffolds, including the tRNA-Sec and a selenocysteine insertion sequence (SECIS) element, which were notable findings in the cranberry mitochondrial genome derived from ‘HyRed’ [71].

### Metabolic pathways

Many KEGG biosynthetic pathways were generated as part of this project. Cranberry is rich in plant secondary metabolites, particularly polyphenolics synthesized from phenylalanine, that not only benefit plant health (e.g., by enhancing disease resistance or deterring herbivores), but also potentially benefit humans. These phytochemicals are in several different biochemical classes, but among the most important are the phenols including the flavonoids. The major flavonoids in cranberry are the proanthocyanidins, anthocyanins and flavonols. All three classes are being intensely investigated for their potential benefit to human health. For example; proanthocyanidins are thought to help in maintaining urinary tract health [7,72], anthocyanins are important as antioxidants [5,73,74] and flavonols are implicated in anti-atherogenic, anti-inflammatory, and anti-cancer bioactivities, among others [75-78].

**Figure 5:**
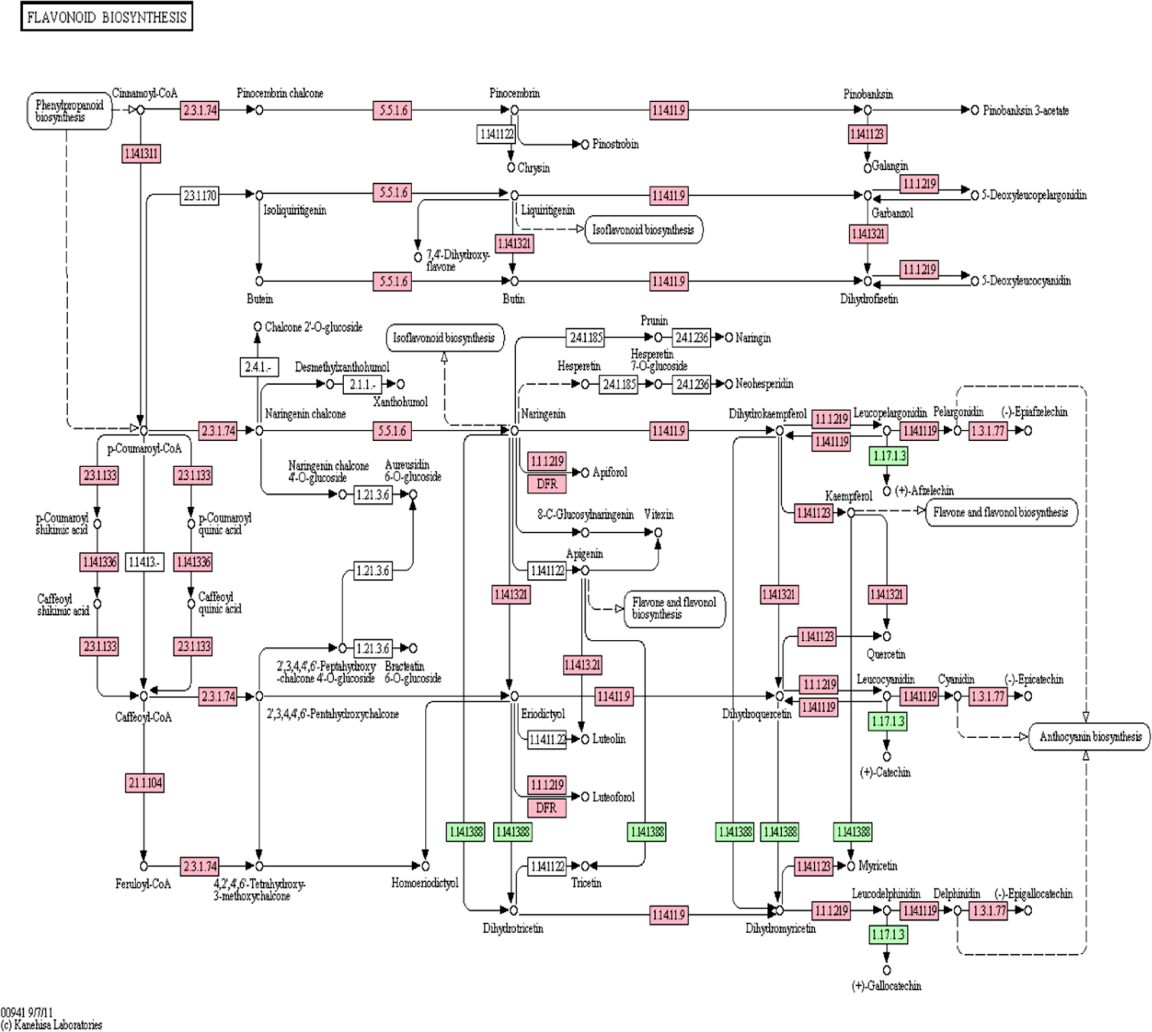
**KEGG reference pathway for flavonoid biosynthesis (map00941).** Enzymes colored in pink were found in both cranberry and grape. Those in green were found in grape, but not cranberry. Those uncolored were found in neither cranberry nor grape.

Considering the importance of the flavonoids, we show the KEGG reference pathway for flavonoid biosynthesis (map00941, Figure 5). The enzymes found in our cranberry sequence are compared to those reported to be found in grape. Our data show that essentially all of those found in grape are also present in cranberry with the exception of two enzymes, flavonoid 3’,5’ hydroxylase (EC: 1.14.13.88) and leucoanthocyanidin reductase (EC: 1.17.1.3). The flavonoid 3’, 5’ hydroxylase (F35H) catalyzes hydroxylation of the B-ring of dihydrokaempferol to form dihydromyricetin. Dihydromyricetin is then converted to blue-colored delphinidins [79]. Thus, F35H catalyzes a key step leading to the biosynthesis of blue pigment in flowers and fruits and is sometimes referred to as the ‘blue’ gene [80]. Ripe cranberries contain primarily the galactosides and arabinosides of cyanidin and peonidin, with small amounts of the glucosides [81,82], resulting in their brilliant red color. Fruit of *V. macrocarpon* are virtually devoid of delphinidin, although interspecific hybrids with *V. oxycoccus* contained trace amounts [15]. It is therefore expected that this enzyme (F35H) might be lacking in American cranberry, but present in purple-blue varieties of grape.

The leucoanthocyanidin reductase catalyzes the synthesis of catechin, catechin-4beta-ol (leucocyanidin) as well as the flavan-3-ols afzelechin and gallocatechin. These compounds and others are important precursors to the biosynthesis of condensed tannins. Although cranberry does contain epicatechin and condensed tannins produced by alternate pathways, catechin, gallocatechin, or afzelechin have not been reported [83,84]. Because cranberry fruit lacks catechin, it is not surprising that a gene encoding the enzyme (leucoanthocyanidin reductase) for its biosynthesis was not found.

Flavonoid biosynthesis is tissue specific, developmentally regulated and can be induced by a variety of environmental factors, including light, UV radiation, fungal infection, interaction with microorganisms, wounding, etc. Many of the ‘core’ structural genes involved in plant flavonoid biosynthesis are known (e.g., [85]). The structural genes and the regulatory genes in this pathway are of interest in cranberry as these affect the temporal and spatial flavonoid biosynthesis as well as the specific flavonoids produced. These genes can be targeted for manipulation of flavonoid biosynthesis through various means or used as markers for selection of desirable flavonoid profiles through breeding. For example, an important aspect of anthocyanins as antioxidants is the specific aglycone, as well as the glycoside, as this affects the both the antioxidant potential and bioavailability [73,86]. Interspecific hybridization was demonstrated to alter anthocyanin glycosylation in cranberry, but the gene(s) involved were not identified [15]. KEGG pathway analysis offers the opportunity to identify these candidate genes, such as the glycosyltransferases, for further study.

Another important class of secondary compounds is the terpenes. Terpenes are a diverse group of compounds that are the primary components of plant essential oils. Their tremendous structural diversity requires a diverse array of enzymes for their synthesis. Terpenes are synthesized via two major pathways; the acetate-mevalonate pathway, which operates in the plant cell cytoplasm and mitochondria, and the non-mevalonate which operates in the plastids [87]. We generated KEGG pathways for biosynthesis of terpenes thought to be involved in plant-insect interactions (not shown). Selected genes encoding key enzymes were identified and used to design real-time PCR primers. These primers were then used to monitor expression of those genes in response to insect feeding [88]. The volatile terpenes produced, as a result of the demonstrated up-regulation of certain genes, deterred further feeding and attracted parasites of the herbivores [88].

## Conclusions

The use of an inbred genotype derived from five generations of selfing, (F ≥ 0.97) where 97% or greater of the alleles are identical by descent, allowed a preliminary assembly of the genome and the transcriptome, and the identification of key genes and gene families in the American cranberry. The data generated not only allow for a myriad of studies of cranberry and related species, but also contribute to the mounting information available on higher plants. These data provide a genomic database of this recently domesticated North American temperate crop, offering facilitation for genetic enhancement, and the study of biotic and abiotic stresses that will be encountered with the changing climate.

## Methods

### Plant material

The cranberry cultivar Ben Lear was selected from the wild in Wisconsin in 1901 [1]. Despite the introduction into cultivation many years ago, ‘Ben Lear’ is still widely grown. In addition to being high-yielding, the fruit of this cultivar ripen relatively early and are deeply colored. ‘Ben Lear’ has been used in breeding programs as a parent, giving rise to the recently released cultivars Crimson Queen and Demoranville [89], and as a grandparent in development of ‘HyRed’ [90]. To reduce heterozygosity, a fifth-generation selfing cycle inbred clone (F ≥ 0.97) of ‘Ben Lear’ designated CNJ99-125-1, was selected for genome sequencing. Genomic DNA was isolated from young expanded leaves of greenhouse-grown ramets of CNJ99-125-1 as described in Georgi et al. [52]. RNA was extracted from greenhouse-grown leaves and tender shoot tips using the RNAqueous kit (Life Technologies, Carlsbad, CA USA) following the manufacturer’s protocol and used for transcriptome sequencing (see below).

### Library construction, sequencing and de novo assembly

The genomic library was prepared using the Paired-End DNA Sample Prep Kit (Illumina, San Diego, CA USA) following the manufacturer’s protocol. Genomic DNA was sequenced on the Illumina Genome Analyzer IIx (GAIIx, 2×150 bp reads); the paired-end library insert size averaged 430 bp. Total RNA was converted into a cDNA fragment library with an average insert size of 354 bp using Illumina’s mRNA-Seq kit (San Diego, CA USA), according to the manufacturer’s protocol; paired-end 2×100 bp reads were also sequenced on the Illumina GAIIx sequencer. Quality trimming and adaptor removal for both DNA and RNAseq reads were done with FASTX-Toolkit (Version 0.6.1). Genomic DNA trimmed reads were assembled with the CLC Genomics Workbench (Aarhus, Denmark). Scaffolding of the assembled contigs was done using SSPACE [91]. cDNA sequences were assembled into ESTs using the CLC Genomics Workbench and ABySS using three different k-mer values (k = 56, 63, 70) [92,93]. Merging the four transcriptome assemblies and removing short redundant contigs was done using BLAT/ CD-HIT-EST [94,95].

### Transposable elements

Transposable elements (TE) in the *V. macrocarpon* assembly were determined using the RepeatMasker tool [96] together with the RepBase database v17.07 [36]. De-novo repeats were modeled using RepeatModeler [97]. The identified TEs were masked from the assembly and the masked assembly was used for all downstream analysis. The data for cucumber, apple, grape, *Arabidopsis*, rice and corn were taken from [98] and the data for melon were taken from [99].

### Gene prediction

Gene model predictions were generated using AUGUSTUS-2.6.1 [35]. AUGUSTUS was trained to be *V. macrocarpon*-specific using 1,000 cDNA assemblies recommended by PASA [100]. The *V. macrocarpon*-specific AUGUSTUS parameters were tested using 1,358 cDNA assemblies recommended by PASA that do not overlap with the 1,000 genes used for training AUGUSTUS. The sensitivity and specificity at the gene level were 0.39 and 0.42, respectively. When running the same set of test genes using the *Arabidopsis* parameters, the sensitivity and specificity were much lower at 0.13 and 0.14, respectively. An increase in sensitivity and specificity at the exon and nucleotide levels were also found when using the *V. macrocarpon*-specific parameters (Additional file 3: Table S2). AUGUSTUS was run on a repeat-masked genome assembly produced by RepeatMasker [96]. The assembled *V. macrocarpon* ESTs and Illumina mRNA-Seq reads were mapped to the genome assembly using GMAP [101] and the resulting mapping was incorporated as a “hint” for AUGUSTUS. The predicted models were compared to Repbase, a transposable element (TE) database [36], by using BLASTP (*e*-value <1E-10), predicted proteins that overlapped >30% with the transposable element (TE) proteins were removed.

### Taxonomic assessment

To verify the taxonomic placement of cranberry, two plastid (atpB, rbcL) and one mitochondrial (matR) gene sequences were concatenated and aligned, using MUSCLE (EMBL-EBI), with those of 18 other plant species including Rosids, Asterids, monocots and a basal eudicot: *Arabidopsis thaliana* (thale cress), *Carica papaya* (papaya), *Citrus sinensis* (orange), *Coffea arabica* (coffee), *Cucumis sativus* (cucumber), *Daucus carota* (carrot), *Glycine max* (soybean), *Gossypium hirsutum* (cotton), *Helianthus annuus* (sunflower), *Lotus japonicus*, *Nicotiana tabacum* (tobacco), *Oenothera elata* (evening primrose), *Ranunculus macranthus* (large buttercup), *Spinacia oleracea* (spinach), *Theobroma cacao* (cocoa), *Vaccinium macrocarpon* (American cranberry), *Vigna radiata* (mung bean), *Vitis vinifera* (grape) and *Zea mays* (corn). The resulting dataset was a 3,596 long nucleotide alignment. The optimal model of DNA substitution for the three genes was the Generalized time reversible model with Gamma distributed among site rate variation and a proportion of invariant sites (GTR + Γ + i) calculated using Modeltest software [102]. A maximum likelihood (RAxML) tree was constructed and the results of 100 bootstrap replicates were used to determine the phylogeny.

### Conserved orthologous set (COSII) markers

Conserved orthologous genes were inferred in cranberry following the methodology of Wu et al. [45]. Two reciprocal best match analyses were performed by comparing cranberry against *Arabidopsis thaliana* (L.) Heynh. and *Lactuca sativa* L., and against *A. thaliana* and *Helianthus annuus* L. cDNA sequences. The two resulting databases were compared to each other using BLASTN with an *e*-value cutoff of 1E-10 to detect the single copy genes in the *V. macrocarpon* transcriptome. Annotation of the obtained COSII markers was performed in BLAST2GO [103].

### Microsatellite detection

Identification of perfect microsatellite sequences or simple sequence repeats (SSRs) was done using the MISA [104] identification tool. The detection was performed in the assembled scaffolds and transcriptome sequences. We included 2-6 bp motifs and repeats with a minimum length of 12 (for di-, tri-, and tetra-nucleotides), 15 (for penta-nucleotides) and 18 (for hexa-nucleotides). Mono-nucleotides were not considered due to the difficulty of distinguishing between a sequencing or assembly error and real repeat sequence variation.

### SNP identification

To determine the SNPs in the *V. macrocarpon* inbred accession, all the genomic reads were mapped back to the assembled scaffolds. The alignments were scanned for SNPs using the CLC Genomics Workbench quality-based variant detection tool with the following parameters: a SNP was called only if it had at least 10X coverage of genomic reads, a minimum Phred quality score of 20 at the position of the SNP and the average quality score of the flanking 5 bp on either side of the SNP was higher than 15. The number of SNPs in the parental ‘Ben Lear’ cultivar was also determined in silico in order to establish the actual degree of homozygosity in the inbred accession relative to the parent.

### Transporter analysis

Identification and classification of transporter proteins in the *V. macrocarpon* predicted proteins data set was done using 6,099 membrane transport protein sequences downloaded from The Transporter Classification Database (TCDB) [105]. To this end, BLASTP with a cutoff *e*-value ≤ 1E-6 was used; sequences with alignment scores less than 100 were filtered out of the data set. To be able to compare between *V. macrocarpon* and *Vitis vinifera,* the same analysis was done on the entire proteome of *V. vinifera* (downloaded from UniProt).

### Identification of transcription factors

Plant transcription factor (TF) protein sequences were downloaded from the Plant Transcription Factor Database v3.0 (PlnTFDB, [106]). Putative *V. macrocarpon* TF were identified using a Reciprocal Blast Hit utilizing BLASTP with a cut-off < 1E-20.

### Putative resistance genes

Identification of putative resistance genes (R-genes) in the *V. macrocarpon* genome was done using homology search. One hundred and twelve manually curated protein sequences of known R-genes, downloaded from the plant R-genes database (PRGdb) [66], were used to search for homologues in the *V. macrocarpon* set of predicted proteins using BLASTP. Sequences with bit scores less than 100 and *e*-values > 1E-10 were removed, in addition, alignments that overlapped less than 60% with their targets were also removed. Putative homologues of genes implicated in *Arabidopsis* immune responses to necrotrophic pathogens (see Additional file 3: Table S2 in [70]) were identified using Reciprocal Blast Hit using BLASTP with a cut-off < 1E-20. The data for cucumber, apple, grape, *Arabidopsis*, rice and corn were taken from [98] and the data for melon were taken from [99].

### Mitochondrial genome comparison

The sequenced mitochondrial (mt) genome from ‘HyRed’ was used as reference [71] to map the scaffolds from the inbred accession of ‘Ben Lear’ assembly. Scaffolds with positive blast hits, with minimum 40 bp length and at least with 80% identity to the ‘HyRed’ mitochondrial sequence were selected for further analysis. Paired end reads with positive hits to the mitochondrial genome were reassembled using ABySS with an optimum k-mer value of k = 58 and the resulting contigs were mapped against the organelle.

### Metabolic pathways

Assigning KEGG Orthologies (KO) to the *V. macrocarpon* predicted proteins and generating the KEGG metabolic pathways were done with KAAS (KEGG Automatic Annotation Server) [107], using the bi-directional best hit method.

## Competing interests

The authors declare that they have no competing interests.

## Authors’ contributions

JP and EZ wrote the paper. DB led the genome sequencing and participated in the bioinformatic analysis of the genome and proteome data with EZ. EZ carried out the bioinformatic analyses including genome assembly, gene prediction, SNP, transporter and KEGG pathway analysis. DF participated in analyzing the sequence data for COS II, SNP and SSR markers and taxonomic assessment, as well as for the detection of organellar scaffolds in the nuclear genome assembly. JZ assisted in data analysis and interpretation, writing and revision of several sections of the manuscript. LG extracted nucleic acids and prepared an mRNAseq library and assisted in revising the manuscript. NV developed the 5^th^ generation selfed accession for sequencing, co-orchestrated the project and contributed to sections of the introduction and discussion. All authors read and approved the final manuscript.

## Supplementary Material

Additional file 1: Table S1 Annotation of 35 conserved ortholog (COSII) genes with known function identified in the American cranberry (*Vaccinium macrocarpon*) transcriptome. Click here for file

Additional file 2: Figure S1 Mapped contigs from reassembled paired end reads to the cranberry mitochondrial genome. Click here for file

Additional file 3: Table S2 Gene prediction sensitivity and specificity at the exon and nucleotide levels when using the *V. macrocarpon*-specific parameters vs. those of *Arabidopsis thaliana*. Click here for file
