# Supplementary material for: The American cranberry: first insights into the whole genome of a species adapted to bog habitat
Source: BMC Plant Biol. 2014 Jun 13;14:165. doi: 10.1186/1471-2229-14-165 (PMC4076063; doi:10.1186/1471-2229-14-165)
Supplement: Additional file 1: Table S1 — Annotation of 35 conserved ortholog (COSII) genes with known function identified in the American cranberry (Vaccinium macrocarpon) transcriptome. [file 1471-2229-14-165-S1.doc]

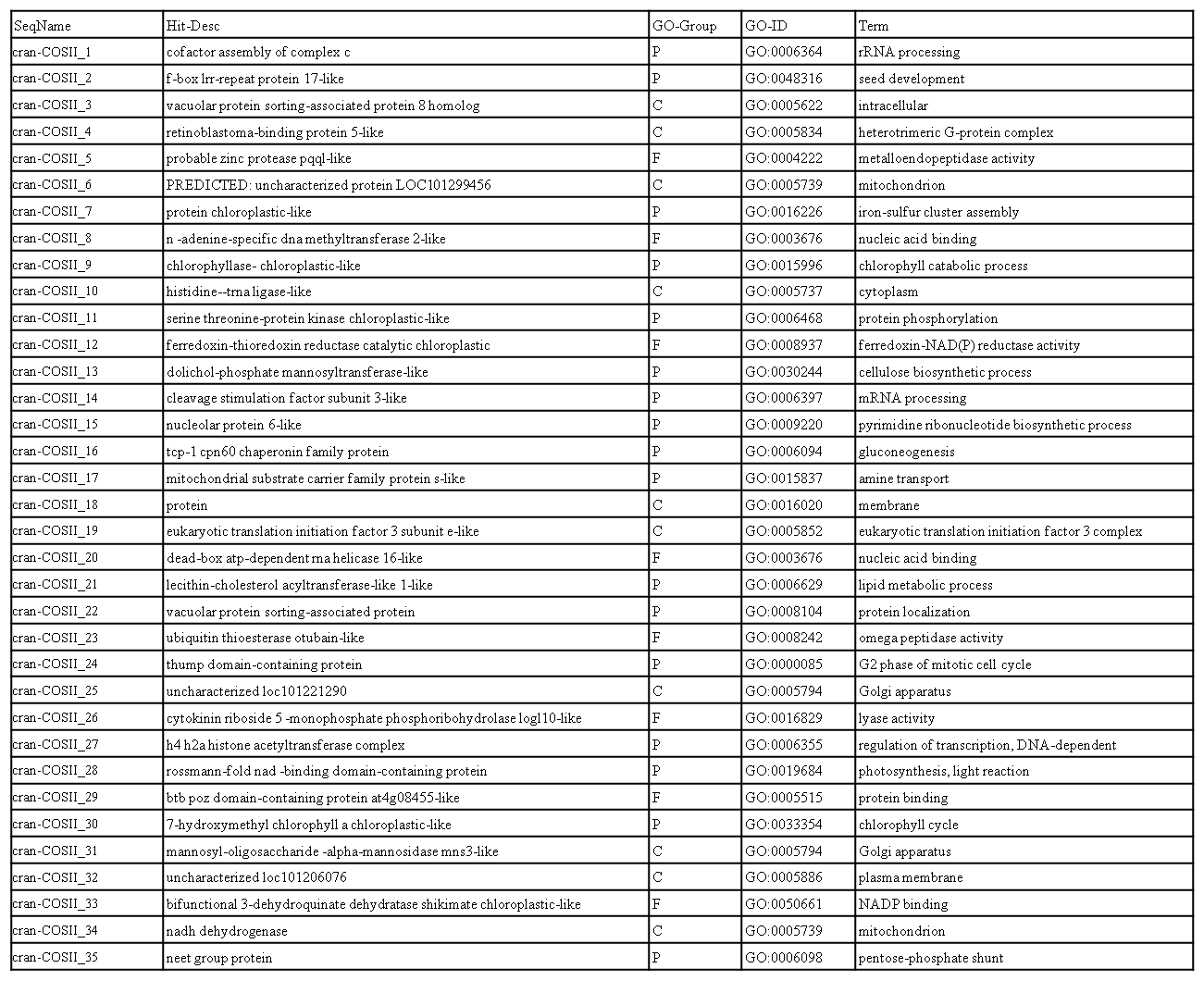


**Supplementary Table 1.** Annotation of 35 conserved ortholog (COSII) genes with known function identified in the American cranberry

(*Vaccinium macrocarpon*) transcriptome.
