# Supplementary material for: The American cranberry: first insights into the whole genome of a species adapted to bog habitat
Source: BMC Plant Biol. 2014 Jun 13;14:165. doi: 10.1186/1471-2229-14-165 (PMC4076063; doi:10.1186/1471-2229-14-165)
Supplement: Additional file 2: Figure S1 — Mapped contigs from reassembled paired end reads to the cranberry mitochondrial genome. [file 1471-2229-14-165-S2.pptx]

## Slide 1
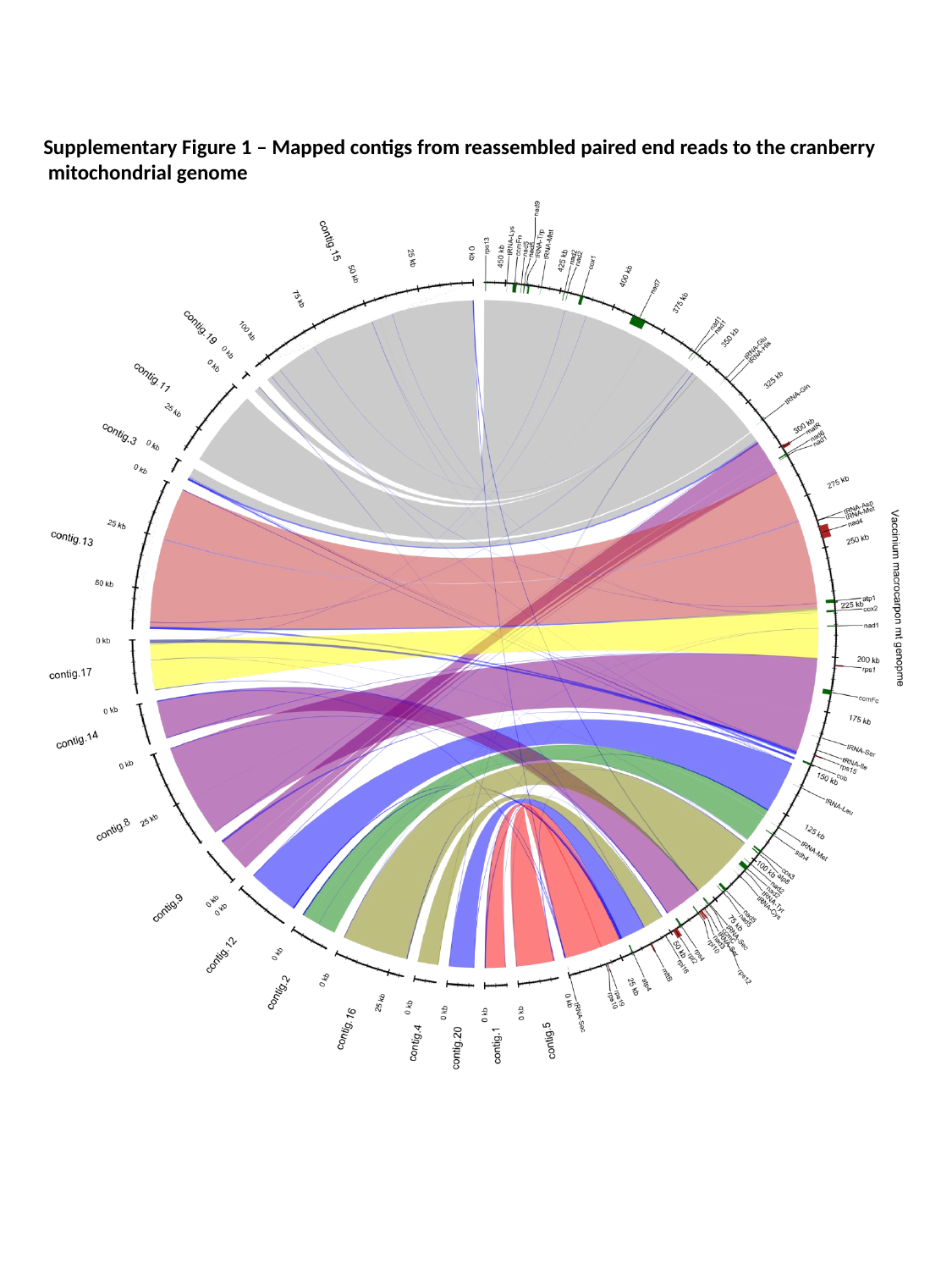

Supplementary Figure 1 – Mapped contigs from reassembled paired end reads to the cranberry
 mitochondrial genome
