# Supplementary material for: The American cranberry: first insights into the whole genome of a species adapted to bog habitat
Source: BMC Plant Biol. 2014 Jun 13;14:165. doi: 10.1186/1471-2229-14-165 (PMC4076063; doi:10.1186/1471-2229-14-165)
Supplement: Additional file 3: Table S2 — Gene prediction sensitivity and specificity at the exon and nucleotide levels when using the V. macrocarpon-specific parameters vs. those of Arabidopsis thaliana. [file 1471-2229-14-165-S3.doc]

**Supplementary Table 2.** Gene prediction sensitivity and specificity at the exon and nucleotide levels when using the *V. macrocarpon*-specific parameters vs. those of *Arabidopsis thaliana*.

|  |  | **Sensitivity** | **Specificity** |
| --- | --- | --- | --- |
| **Nucleotide level** | *V. macrocarpon* | **0.9** | **0.95** |
|  | *A. thaliana* | 0.9 | 0.91 |
| **Exon level** | *V. macrocarpon* | **0.74** | **0.8** |
|  | *A. thaliana* | 0.67 | 0.64 |
| **Gene level** | *V. macrocarpon* | **0.39** | **0.42** |
|  | *A. thaliana* | 0.13 | 0.14 |
